# Emergency Department Length of Stay for Maori and European Patients in New Zealand

**DOI:** 10.5811/westjem.2016.5.29957

**Published:** 2016-06-21

**Authors:** David Prisk, A. Jonathan R. Godfrey, Anne Lawrence

**Affiliations:** * Palmerston North Hospital, Mid Central Health, Emergency Department, Palmerston North, New Zealand; † Massey University, Department of Statistics, Palmerston North, New Zealand

## Abstract

**Introduction:**

Emergency department length of stay (ED LOS) is currently used in Australasia as a quality measure. In our ED, Maori, the indigenous people of New Zealand, have a shorter ED LOS than European patients. This is despite Maori having poorer health outcomes overall. This study sought to determine drivers of LOS in our provincial New Zealand ED, particularly looking at ethnicity as a determining factor.

**Methods:**

This was a retrospective cohort study that reviewed 80,714 electronic medical records of ED patients from December 1, 2012, to December 1, 2014. Univariate and multivariate analyses were carried out on raw data, and we used a complex regression analysis to develop a predictive model of ED LOS. Potential covariates were patient factors, temporal factors, clinical factors, and workload variables (volume and acuity of patients three hours prior to and two hours after presentation by a baseline patient). The analysis was performed using R studio 0.99.467.

**Results:**

Ethnicity dropped out in the stepwise regression procedure; after adjusting for other factors, a specific ethnicity effect was not informative. Maori were, on average, younger, less likely to receive bloodwork and radiographs, less likely to go to our observation area, less likely to have a general practitioner, and more likely to be discharged and to self-discharge; all of these factors decreased their length of stay.

**Conclusion:**

Length of stay in our ED does not seem to be related to ethnicity alone. Patient factors had only a small impact on ED LOS, while clinical factors, temporal factors, and workload variables had much greater influence.

## INTRODUCTION

Racial disparities in emergency care have been well documented. Most investigations of racial disparities in emergency care have occurred in the United States and have addressed differences in care provided to whites as compared to black and Hispanic patients. In general, non-white patients receive less evaluation and treatment for acute conditions, and non-white patients spend longer waiting in the emergency department (ED), both before and after evaluation by an emergency physician.

In 2009, one study found that black patients admitted to hospital (intensive care unit [ICU] and non-ICU) through the ED have longer ED length of stay (LOS) compared to non-blacks.^1^ In 2013, another study found that pediatric non-Hispanic black and Hispanic patients were less likely to receive any analgesic or narcotic analgesic, and were more likely to have a prolonged ED LOS than non-Hispanic white patients who presented with abdominal pain.^2^ In an analysis of ED wait times for stroke patients in the U.S. in 2011, black patients had longer wait times than Hispanic or white patients, with the suggestion that this led to treatment delays and sub-optimal stroke care.^3^ Sonnenfeld, et al. in 2012 also found that non-Hispanic black patients wait longer for ED care than whites,^4^ while in Australia in 2009, Brown and Furyk found that although “there was no statistically significant disparity based upon race in the management of minor head injuries,” indigenous patients waited longer to be seen.^5^

In Australasia, ED LOS is currently used as a quality measure, as increased LOS has been associated with ED crowding, longer inpatient lengths of stay, and increased mortality.^6^ In New Zealand, there is a strong drive to ensure that 95% of patients are admitted, discharged, or transferred from an ED within six hours;^7^ in Australia, the National Emergency Access Target is for 90% of patients to have their ED visit completed within four hours.^8^ As part of a demographic audit of our provincial New Zealand ED, it was noted that Maori, the indigenous people of New Zealand, had a significantly shorter ED LOS than European patients. This came as some surprise, as Maori are well documented to have poorer health outcomes overall.^9^ We therefore sought to investigate the drivers of LOS in our ED, particularly looking at ethnicity as a determining factor.

## METHODS

### Practice Setting

This study took place in the ED of a provincial hospital located on the lower North Island of New Zealand, with a city population of approximately 80,000 people and a total catchment population of around 165,000 people. Approximately 18% of this region’s population identify as Maori (about 15% of New Zealand’s total population of 4.5 million people identify as Maori). To be considered “Maori” in New Zealand, you must identify culturally as being Maori and be able to identify a whakapapa, or family lineage. “Europeans,” generally those people who identify as being of northern European ancestry and commonly referred to as “white” or “Caucasian” in North American literature (and sometimes referred to as Pakeha in Maori literature), make up the great majority (just under 80%) of the rest of our region’s population. Pacific Islanders (people from 22 island nations, of widely varying ethnicities and cultures) comprise about 2% of our region’s and our ED’s population, and Asians (defined broadly as being from India, Southeast Asia, Indonesia, Malaysia, China, or Japan) make up about 10% of our region’s population and about 3% of the ED population. An ill-defined “Other” category encompasses people from Africa, Central and South America, and the Middle East; these people make up approximately 0.3% of our total population.

Annual ED volumes in our hospital total about 40,000 patients per annum. Our ED has 16 beds (two large resuscitation bays, four smaller resuscitation beds, and 10 assessment beds). We have a four-bed minor works station and a five-bed, three-chair observation unit. In our group there are nine consultants (attending emergency physicians), 18 resident medical officers (RMOs) (typically four emergency medicine registrars and 14 post-graduate year two and above senior house officers (SHOs) who are not in a specialty training program and are typically in the ED for only three months), and one permanent and several locum Medical Officers of Special Scale (doctors who have not completed a training program in emergency medicine but may have trained in general practice and are significantly more senior than RMOs). Two clinical nurse specialists staff our Minor Works Station from 11am – 9pm on most days, and they see triage category 4 and 5 patients with minor extremity injuries. One or two consultants and two RMOs work clinically from 7am – 5pm, another consultant and two more RMOs work 12pm – 10pm, and another consultant and another two RMOs work 2pm – 12am. Overnight, a consultant is on call from home, and the ED is staffed by three RMOs (as often as possible, one registrar and two SHOs). General practitioners (GP) will also directly refer patients to medical or surgical specialty registrars; many of these patients will often have at least a partial outpatient workup completed and will be seen only by the non-emergency medicine registrar. Our junior doctors have six-year undergraduate medical degrees from New Zealand, the United Kingdom, or Australia. We are an Australasian Level IV trauma center (roughly equivalent to a Level II trauma center in the U.S.). For the study period, 36.68% of all patients presented by ambulance, 63.24% presented through our front reception/triage area, and 0.07% presented by helicopter.

### Study Design

This was a retrospective cohort study that reviewed 80,714 electronic medical records (PIMS, or Patient Information Management System, Avant Version 2.31, written by Adrian Hunter, copyright 1999–2012) of ED patients from December 1, 2012, to December 1, 2014 (inclusive). All data were collected in March 2015. For this study, we used total ED LOS, as data regarding waiting times to see a nurse or doctor, as well as referral times to specialties, were found to be inconsistent and unreliable.

The decision was made to exclude from the analysis Asian and Pacific Islander patients, as well as patients defined as being “Other” and those patients who did not state an ethnicity. We felt that these categories were too broad and ill-defined, while European and Maori definitions/identity were relatively firm.

We carried out univariate and multivariate analyses on the raw data, and a complex regression analysis was used to develop a predictive model of ED LOS. A subset of 80,029 records with complete data for all variables was used in model development. We initially performed exploratory graphs, t-tests, contingency tables, and chi-squared tests of association to explore associations between pairs of variables. Poisson regression analysis was used to model the effect of temporal factors on patient numbers. Log transformed LOS (log (LOS + 1 minute) was used to change the raw data to a more normal distribution in order to meet the assumptions of our linear regression model. We used regression trees to model LOS, and classification trees were used to model LOS greater than six hours. In the modeling process, we removed variables from the model if they did not improve the model. Akaike information criterion (AIC) was used to compare models. Statistical significance was taken as p-value<0.05.

The primary outcome measure was ED LOS. Potential covariates were broken down into four large categories: patient factors (age, gender, socioeconomic deprivation level (see Appendix 1 for an explanation of the New Zealand Deprivation Index), and ethnicity); clinical factors (presenting complaint, Australasian Triage Scale category, disposition/outcome, seniority of ED staff caring for the patient, identification of a GP (general practitioner), and diagnostic tests performed); temporal factors (hour of day, day of week, month of year); and workload variables (the volume and acuity of patients presenting three hours before and two hours after the arrival of any given patient) were all considered in developing a model to predict ED LOS.

Initially, the date a patient filled a prescription written in the ED was examined as a clinical factor, but records were found to be sparse and unreliable. Therefore, we excluded prescription data from the analysis.

We also found data about time to triage, time to be placed in an ED bed/to be seen by an ED nurse, time to be seen by a doctor, and time of referral to an inpatient specialty to be inconsistently recorded. Therefore, these data were also excluded from the analysis.

Repeat visits by the same patient were not excluded.

We used patient factors and temporal factors to investigate any potential bias in the assignment of Australasian Triage Scale (ATS) category.

Categorization of presenting complaint was done manually, resulting in 62 categories with at least 90 patients in each, including a large “other” category with 31,723 patients.

To evaluate the impact of patient volume and acuity on ED LOS, we created four workload variables to account for the number of patients who presented to the ED in the three hours prior or two hours after the arrival of a baseline patient.

We performed the analysis using R studio 0.99.467 with the packages ggplot2 1.0.1, knitr1.11, lattice 0.20-33, rattle 3.5.0, rcolorBrewer 1.1-2, rpart 4.1-9, rpart.plot 1.5.3 and xtable 1.7-4. R studio is available from RStudio Team (2015); all packages are available from CRAN at http://CRAN.R-project.org/.

In summarizing the medical literature, the decision was made to keep the ethnic terms used in the original papers; most commonly, in American literature, “black” and “Hispanic” were used instead of “African-American” or “Latino.” It should be noted that in New Zealand the terms “black” and “white” are generally unacceptable.

Ethical approval was received from the New Zealand Ethics Committee, and local approval for the study was received from our District Health Board’s chief medical officer, operations manager, and clinical director of the ED. Formal consultation was also sought with the Director of Maori Health.

## RESULTS

### Raw Data

During our study period, 60,601 Europeans (75.1% of the total) and 13,939 Maori (17.3%) presented to our ED. There were 40,300 females and 40,411 males. Most patients (39,138) were triage category 3, and of all ATS categories, these patients had the longest average ED LOS (346.8 minutes).

Mean LOS for all patients was 302.9 minutes. European patients had a mean LOS of 315.9 minutes, while Maori patients had a mean LOS of 266.8 minutes. Mean age of European patients was 46.85 years, and mean age of Maori patients was 29.89 years. See Appendix 2 for more raw data.

### Baseline Patient

For the predictive model, a baseline “average” patient was created from the data: a European male aged between 20 and 50 years with socioeconomic deprivation level 9 who presented with “other” as chief complaint at 11 am on a Monday in January 2013. This patient was ATS category 3, identified a GP, was seen by an ED SHO, had bloodwork and radiographs done, and was discharged home at the conclusion of his ED visit. In our model, there were no patients who presented to the ED in the previous three hours or subsequent two hours relative to his presentation time. The LOS for this baseline patient was 171.8 minutes.

### Patient Factors

Ethnicity dropped out in the stepwise regression procedure. Cohorts were not the same independent of ethnicity. The regression model identified which variables were significant predictors of LOS in the modeling process; variables were removed from the model if they did not significantly improve the model. When patient factors of age, gender, and deprivation score were taken into account, ethnicity did not have a statistically significant effect on LOS; in light of the demographic variables other than ethnicity, there was not independence between ethnicity and the other patient factors.

**Figure 1:**
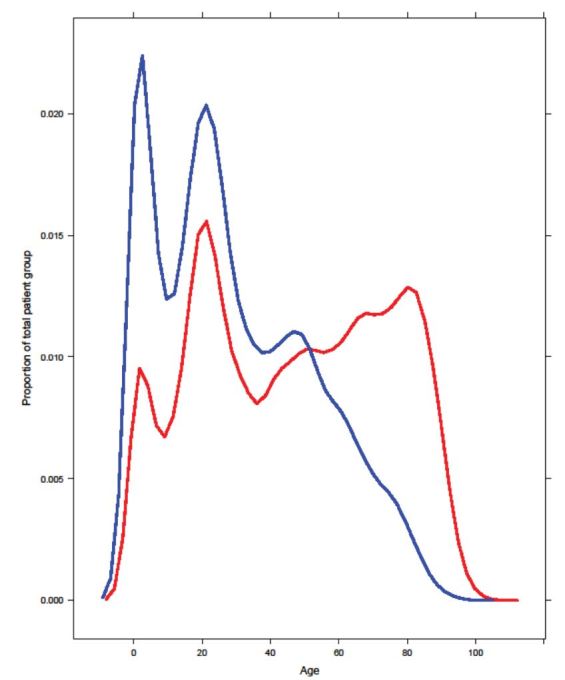
Age distribution among ethnic groups: Maori were statistically younger than Europeans. Average age of 13,939 Maori patients was 29.89 years and average age of 60,601 European patients was 46.85 years.

Age distribution varied across ethnic groups, with Maori overrepresented in the younger age groups and underrepresented in the older age groups compared to Europeans (Figure 1). Patients were broken into five age groups (under two years old, 2–20, 20–50, 50–80, and over 80), and distribution of ED LOS within age groups was found to be similar for different ethnic groups. Average LOS increased with increasing age. Within the five age groups, Maori patients had significantly longer average ED LOS than Europeans for patients 0–2 years old and over 80 years old. Maori patients 20–50 years old and 50–80 years old also had a longer ED LOS than Europeans, but this difference did not reach statistical significance (p=0.059 and p=0.75, respectively). There was no signficant interaction between age and ethnicity. We found that having adjusted for age across ethnic groups equally, making an adjustment for the age of Maori patients was not relevant or necessary.

In general, younger patients had shorter expected LOS, but the relationship was not simple, as the effect of age depended on gender; the interaction between gender and age was statistically significant (Table 1).

Maori patients were more likely to have higher deprivation scores (were more deprived) and the effect of deprivation level was independent of other factors. ED LOS of deprivation levels 7,8, and 10 were not significantly different to that of baseline deprivation level 9. However, deprivation levels 1–6 had significantly shorter ED LOS compared to baseline (Table 2).

### Clinical Factors

Clinical factors had greater impact on ED LOS than patient factors.

We could not predict ATS category from patient factors (age, gender, ethnicity, deprivation level) or temporal factors (hour of day, day of week, month of year).

In our predictive model, the seniority of ED staff caring for the patient had a significant impact on their expected LOS. The greater the experience of the ED doctor, the shorter the expected LOS for the patient (Table 3).

Maori patients were more likely than Europeans to be seen only by a nurse, and were more likely to be discharged home and to self-discharge. Outcome/disposition had a significant and sizeable effect on LOS. Patients sent to an on-site outpatient specialty clinic had the shortest expected ED LOS, while those who were admitted as an inpatient, were transferred to another hospital, or died in ED remained longest in the department (Table 4).

**Figure 2:**
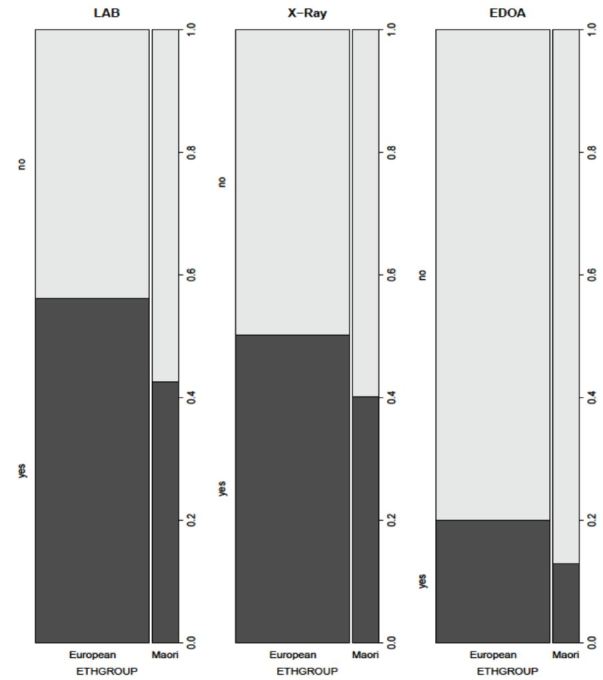
Proportion of Maori and European patients admitted to our emergency department observation area (EDOA), had blood tests performed (Lab), or had radiographs performed.

Maori patients were less likely than Europeans to have labs and radiographs done, and less likely to go to our ED observation area (Figure 2). Not having lab tests done while in the ED decreased LOS by about 30% from baseline, while not having radiographs decreased LOS by about 20% from baseline. Lab tests and radiographs were strongly interdependent, with patients who had lab tests being more likely to have radiographs, and vice versa. The clinical factor with the biggest impact on ED LOS was going to the ED observation area (EDOA), which approximately doubled expected LOS. Patients who breached the six-hour target were more likely to go to EDOA than those patients who did not breach.

Additionally, patients identifying a GP had statistically significant but minimal impact, increasing predicted ED LOS by about 3%. See Table 5 for LOS estimates by EDOA, lab, radiograph, and GP.

Some presenting complaints had statistically significant impact on expected ED LOS. However, the impact tended to be minor compared to the impact of other clinical variables. The largest effects were from diarrhea and vomiting (increased LOS by nearly 30%), crisis (psychiatric evaluation) (increased LOS by 28%), overdose (increased LOS by 25%), toothache (decreased LOS by 15%), SVT (supraventricular tachycardia) (decreased LOS by 12%), and palpitations (decreased LOS by 12%) (Table 6). See Appendix 3 for table of presenting complaint by ethnicity.

Triage category had a significant impact on expected LOS, with triage 1 patients having the shortest expected ED LOS and triage 3 patients having the longest (Table 7).

### Workload Variables

Workload variables had a statistically significant influence, and their impact on ED LOS was complex. Patients who were triaged as less urgent than our baseline patient had only a small impact on our baseline patient’s expected LOS (regardless of whether they arrived before or after that patient). However, every more urgent patient who arrived before our baseline patient was associated with an increase in that patient’s expected LOS.

### Temporal Factors

Temporal factors (hour of day, day of week, month of year) had a significant impact on LOS even when patient volume and acuity were taken into account. Expected ED LOS followed a roughly cyclical pattern during the day, with a peak at 11 am and a low at 4 pm. Expected LOS was shortest on Fridays and greatest on Sundays, shortest in January (summer) and longest in August (winter). Day alone was not a statistically significant predictor of ED LOS, but some combinations of day and month were significant. Time of day had more impact on patient numbers during the weekend than on weekdays. The effect of days of the week and months of the year had a small impact compared to differences across hours of the day.

**Figure 3:**
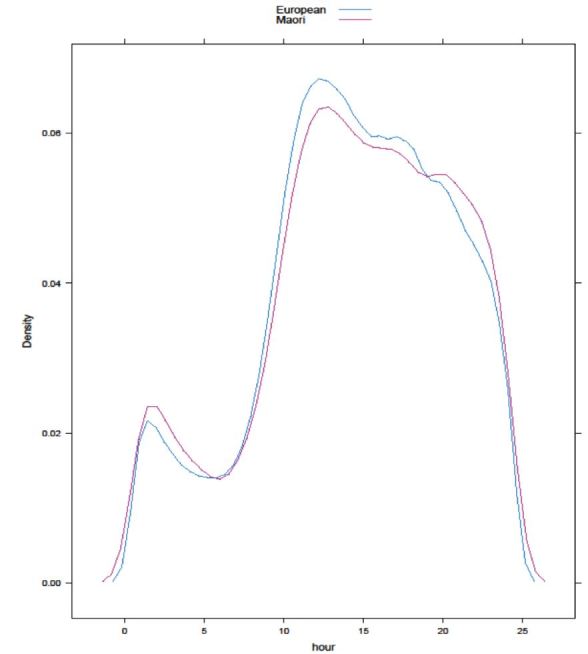
Hourly presentation patterns to the emergency department by ethnicity.

Hourly and daily presentation patterns of different ethnicities were not significantly different (please see Figure 3 for hourly presentation patterns by ethnicity), but monthly presentation patterns were significantly different by group.

### Summary

Overall, there was longer ED LOS on average for women, older people, patients presenting during the middle months of the year (June-August), patients presenting late at night, patients seen by junior doctors, and for ATS category 2 and 3 patients. Although older age, female gender, and greater socioeconomic deprivation level had statistically significant effects on ED LOS, this effect was small compared to clinical factors, temporal factors, and workload variables. After controlling for other factors, ethnicity was not a statistically significant predictor of length of stay.

## DISCUSSION

Our study reinforces some previous findings about ED LOS. A recent systematic review of ED LOS studies found that admission, older age, diagnostic testing, and moderate acuity were related to longer LOS.^10^ In a study of American patients with psychiatric illness, ED LOS was prolonged not only by alcohol on toxicology screening, but by older age, being uninsured (potentially a marker of socioeconomic deprivation), and diagnostic imaging.^11^ Also similar to our study, Kocher et al. in 2012 found that discharged patients had a shorter ED LOS than admitted patients, and that blood tests and advanced imaging significantly prolonged LOS, particularly among discharged patients.^12^

Similar to the findings in our paper, Payne and Puumala found that nonwhite (Native American, African American, Hispanic, or biracial) children were also less likely to receive laboratory and radiological testing.^13^ Hambrook in 2010 found that white, insured American pediatric patients who presented with chest pain were significantly more likely to receive diagnostic testing.^14^

In 2010, using quantile regression to analyze data from four academic EDs in the U.S., Ding and colleagues found that triage level 3 patients waited the longest, and temporal factors had the greatest impact on their waiting times; they also found that temporal factors were strong predictors of service completion times.^15^ Ding also found that patient characteristics had minimal influence on ED service completion time, although ethnicity/race was not included as a demographic feature.^15^ Ding also found that ED volume was highest in the late morning and afternoon hours and lowest at night and in the early morning hours.^15^ In keeping with the findings of our study, and similar to findings of a study in Australia,^16^ the study by Fee et al. in 2012 also found that junior doctors were associated with prolonged ED LOS for admitted, discharged, and transferred patients compared to senior emergency physicians.^17^ However, Fee, in this same study, found that nonwhite race was associated with longer ED LOS among admitted patients,^17^ and Bekmezian et al found that Hispanic ethnicity (as well as winter season and early morning arrival) were associated with prolonged ED LOS.^18^ This association of ethnicty with prolonged ED LOS is somewhat different to what we found.

While our study did not find an association between ethnicity and ED LOS, in 2007 Gardner et al. found that advanced imaging and Hispanic ethnicity were independently associated with longer LOS; also unlike our study, emergency physician seniority did not impact significantly on ED LOS.^19^ Mansbach et al. also found that Hispanic race/ethnicity in children with bronchiolitis was associated with increased ED LOS.^20^ Unlike our findings, racial disparities in ED triage scoring have also been found in more recent papers.^21^–^23^

One finding that seems to be consistent across several papers over several years is that nonwhite (especially indigenous) and socioeconomically deprived patients are more likely to leave the ED before the completion of evaluation and treatment.^24^–^27^ We, too, found this to be the case in our study.

LOS in our ED does not seem to be directly related to ethnicity alone. Among other factors, the age distribution of Maori patients is very different to that of European patients; Maori in general have an average life expectancy 7.1 years less than non-Maori patients,^28^ and this is reflected in their age distrubtion in our ED. The age structure of the Maori population nationwide is also heavily skewed toward younger people.^29^ When age group is taken into account, Maori do not have a shorter average LOS than European patients; at the extremes of age, the average LOS is significantly longer for Maori.

Additionally, females in New Zealand have a life expectancy 3.7 years higher than males,^28^ which may partially account for their increased LOS.

Deprivation level was a significant confounder, as it was an independent predictor of LOS.

Clinical factors were of greater significance than patient factors, with the biggest influence on LOS being whether a patient went to our ED observation area or not. Interestingly, many patients who had already been in the ED for six hours or more were more likely to go to EDOA. This suggests that EDOA was perhaps being used as a surrogate inpatient ward for admitted patients during the study period, and that there was impeded flow into or through the hospital.

Other important clinical factors that significantly impacted on ED LOS were triage category, the seniority of the doctor seeing the patient, disposition, and whether or not labs or radiographs were obtained. As ATS category appeared to be unpredictable by patient factors or temporal factors, it could reasonably be argued that ATS category was an objective evaluation of patient acuity. This, in turn, suggests that the seniority of practitioner seeing the patient, the need for bloodwork, radiographs, and observation or admission were also not subject to significant bias, although these were not specifically tested. Maori were less likely to identify a GP, less likely to have radiographs or blood tests, and more likely to be discharged and to self-discharge, all factors which decreased their LOS.

In our predictive model, Maori were less likely to go to EDOA, one of the biggest extenders of ED LOS, but this may be related to their younger age; they could reasonably be assumed to have fewer comorbidities, although information about comorbidities was not available to us and this lack of information might have introduced significant bias.

Workload variables had an important and complex impact on LOS for all patients. Both volume and acuity were important before and after any given patient’s arrival. However, hour of day, day of week, and month of year also had a significant effect on ED LOS, even when workload variables were taken into account. This suggests that the predictable hourly, daily, and seasonal variation as well as the unpredictable viscosity (volume *and* acuity) of the ED have significant impacts on LOS for any patient.

## LIMITATIONS

We did not have access to comorbidities, as these were not recorded electronically. Comorbidity would be an important variable to include in any future study of ED LOS. We also found data about time to triage, time to be placed in an ED bed, time to be seen by a doctor, time to be seen by an ED nurse, and time of referral to an inpatient specialty to be inconsistently recorded; these times, too, would be important to include as additional variables in a future study, as they might uncover important disparities not reflected in total ED LOS. Total ED LOS might not have been attributable to physician or departmental factors.

As a retrospective study on all patients presenting over a given time period, the cohorts were not matched. We recognize that cohort matching is desirable but it was not a requirement for our model. Aside from the practical difficulties, not knowing which factors were significant for LOS made matching cohorts problematic. We did not try to make predictions for any ethnic group or the differences among them because after adjusting for other factors, ethnicity became irrelevant.

Patients who self-discharged - or were discharged home by a nurse - were not of equal severity and cohort and this, too, was a limitation.

Presenting complaint was a problematic category, as it could be recorded as free text by a triage nurse, a staff nurse, a charge nurse, or a receptionist (without a medical background). Including presenting complaint added even more complexity to our model, without significantly improving it. Most presenting complaints did not seem to affect LOS in a significant way.

Ideally, this model could be applied in a multicenter review that would include similar-sized EDs within New Zealand.

## CONCLUSION

There were many confounders in determining length of stay in our emergency department. It would seem that social issues (perhaps including access to primary care) and calendar events outside the ED, as well as unpredictable workload variables within the ED, strongly impact on ED length of stay for all patients. Why do Maori have a shorter ED LOS? They are younger, less likely to have a GP, and less likely to receive blood tests, radiographs, be admitted, or go to EDOA; they are also more likely to be discharged and to self-discharge. *Why* these things are so is an open question and provides direction for further study.

## Supplementary Information







## Figures and Tables

**Table 1:** Estimated length of stay (LOS) by age and gender (all other factors at baseline).

Age	Male LOS (minutes)	Female LOS (minutes)
0–2 yo	163.0 (CI 153.2–173.3)	156.0 (CI 146.6–166.0)
2–20 yo	161.5 (CI 152.3–171.2)	170.4 (CI 160.7–180.6)
20–50 yo	171.8 (CI 162.2–181.9)	180.7 (CI 170.6–191.3)
50–80 yo	178.9 (CI 169.0–189.5)	185.6 (CI 175.3–196.5)
80–110 yo	185.1 (CI 174.4–196.5)	193.0 (CI 181.9–204.7)

**Table 2:** Estimated LOS by socioeconomic deprivation level (all other factors at baseline). Level 1 is least deprived and 10 is most deprived; see Appendix 1 for details of the New Zealand Deprivation Index.

Socioeconomic deprivation level (from least to greatest)	ED LOS (minutes)
1	163.4
2	162.9
3	167.4
4	166.6
5	165.2
6	168.5
7	169.6
8	171.9
9	171.8
10	173.0

*LOS,* length of stay

**Table 3:** Estimated length of stay (LOS) by practitioner (all other factors at baseline).

Category of practitioner	LOS (minutes)
ED registered nurse (RN)	78.7 (CI 74.1–83.7)
Emergency clinical nurse specialist (CNS)	115.1 (CI 108.2–122.3)
Other specialty registrar	123.3 (CI 116.2–130.8)
Consultant emergency physician	148.9 (CI 140.3–157.9)
ED medical officer special scale (MOSS)	153.2 (CI 143.8–163.1)
Emergency medicine registrar	162.6 (CI 153.4–172.3)
ED senior house officer (SHO)	171.8 (CI 162.2–181.9)

*ED,* emergency department

**Table 4:** Length of stay (LOS) estimates by outcome/disposition (all other factors at baseline).

Outcome/disposition	LOS (minutes)
On site specialty clinic	121.9 (CI 112.9–131.6)
Discharged home	171.8 (CI 162.2–181.9)
Self discharge	172.0 (CI 161.7–183.0)
Mental health emergency team	199.7 (CI 167.3–238.3)
Admitted as inpatient	219.0 (CI 206.8–232.0)
Transfer to another hospital	231.7 (CI 209.6–256.1)
Deceased	314.7 (CI 269.0–368.1)

**Table 5:** Length of stay estimates in minutes according to emergency department observation area, lab, radiograph and GP-known or not (all other factors at baseline).

	Admission to emergency department observation area	Lab (blood tests performed)	X-ray performed	General practitioner known
Yes	348.0	171.8	171.8	171.8
No	171.8	119.0	137.5	166.5

*GP,* general practitioner

**Table 6:** Presenting complaints that significantly increased or decreased length of stay (LOS) from baseline.

Presenting complaint	LOS (minutes)
Diarrhoea and vomiting	240.1
For crisis (psychiatric evaluation)	223.9
Diarrhoea	218.1
Overdose	209.9
Palpitations	149.0
SVT (supraventricular tachycardia)	147.7
Toothache	146.2

**Table 7:** Length-of-stay (LOS) estimates according to triage level (other factors at baseline). Level 1 is most acute; level 5 is least acute.

Australasian triage scale category	LOS (minutes)
1	99.4
2	151.5
3	171.8
4	154.7
5	108.3
