# Supplementary material for: Emergency Department Length of Stay for Maori and European Patients in New Zealand
Source: West J Emerg Med. 2016 Jun 21;17(4):438–48. doi: 10.5811/westjem.2016.5.29957 (PMC4944800; doi:10.5811/westjem.2016.5.29957)
Supplement: Supplementary file 1 [file wjem-17-438-s001.docx]

Appendix 1

New Zealand Index of Deprivation 2013

“NZDep2013 combines 9 variables from the 2013 census which reflect 8 dimensions of deprivation. NZDep2013 provides a deprivation score for each meshblock in New Zealand. Meshblocks are geographical units defined by Statistics New Zealand, containing a median of approximately 81 people in 2013.”

“The NZDep2013 index of deprivation ordinal scale ranges from 1 to 10, where 1 represents the areas with the least deprived scores and 10 the areas with the most deprived scores.”

“The NZ Dep 2013 scale of deprivation from 1 to 10 divides New Zealand into tenths of the distribution of the first principal component scores. For example, a value of 10 indicates that the meshblock is in the most deprived 10 percent of areas in New Zealand…”

“The NZDep2013 deprivation scores apply to areas rather than to individual people.”

“NZDep2013 combines the following census data (calculated as proportions for each small area):”

| **Dimension of Deprivation** | **Description of variable (in order of decreasing weight in the index)** |
| --- | --- |
| Communication | People aged < 65 with no access to the internet at home |
| Income | People aged 18-64 receiving a means tested benefit |
| Income | People living in equivalised households with income below an income threshold |
| Employment | People aged 18-64 unemployed |
| Qualifications | People aged 18-64 without any qualifications |
| Owned home | People not living in own home |
| Support | People aged < 65 living in a single parent family |
| Living space | People living in equivalised households below a bedroom occupancy threshold |
| Transport | People with no access to a car |

All of the above information taken from:

**Atkinson J, Salmond C, Crampton P.** NZDep2013 Index of Deprivation User’s Manual. Department of Public Health, University of Otago, Wellington. May 2014; pp 1-6.
