# Supplementary material for: Emergency Department Length of Stay for Maori and European Patients in New Zealand
Source: West J Emerg Med. 2016 Jun 21;17(4):438–48. doi: 10.5811/westjem.2016.5.29957 (PMC4944800; doi:10.5811/westjem.2016.5.29957)
Supplement: Supplementary file 2 [file wjem-17-438-s002.docx]

Appendix 2

Raw Data

ED LOS increased with increasing age (Table 1).

|  | **0-2 years old** | **2-20 years old** | **20-50 years old** | **50-80 years old** | **80-110 years old** |
| --- | --- | --- | --- | --- | --- |
| **LOS (minutes)** | 170.6 | 210.7 | 273.2 | 369.2 | 459.9 |

Table 1: Mean ED length of stay by age group

ED length of stay increased with increasing deprivation level, and Maori were overrepresented in higher deprivation levels. ATS category became more acute with increasing socioeconomic deprivation and with increasing age. Maori patients were overrepresented in ATS categories 1, 4, and 5. ATS category 5 patients had the shortest ED LOS and ATS category 3 patients had the longest. Maori patients had a significantly less acute average triage score than Europeans (tables 2-5).

| **ATS category** | **European (% total ATS category)** | **Maori** | **Mean Age** | **Mean Deprivation** | **Mean ED LOS (IQR)** |
| --- | --- | --- | --- | --- | --- |
| **1** | 314 (73.5%) | 89 (20.8%) | 51.2 | 7.00 | 221.2 (94-272) |
| **2** | 6144 (76.5%) | 1322 (16.5%) | 53.1 | 6.88 | 332.0 (182-393) |
| **3** | 29 901 (76.4% | 6558 (16.8%) | 44.86 | 6.84 | 346.8  167-419) |
| **4** | 21 393 (73.6%) | 5171 (17.8%) | 38.17 | 6.73 | 259.9 (110-318) |
| **5** | 2743 (70.7%) | 759 (19.6%) | 34.23 | 6.65 | 142.4  (59-191) |

Table 2: Demographics by ATS category

.

| **Outcome/Disposition** | **Mean Age (years)** | **Mean Deprivation Score** | **Mean ED LOS (minutes)** | **Mean ED Triage Score** |
| --- | --- | --- | --- | --- |
| **Admitted as Inpatient** | 53.71 | 6.91 | 377.0 | 2.95 |
| **Clinic** | 29.36 | 6.88 | 193.8 | 3.38 |
| **Deceased** | 66.16 | 6.72 | 306.2 | 1.59 |
| **Did Not Wait** | 17.00 | 7.00 | 1.0 | N/A |
| **Discharged Home** | 38.87 | 6.75 | 282.1 | 3.50 |
| **MHET (Mental Health Emergency Team)** | 35.58 | 7.13 | 309.8 | 3.22 |
| **Self Discharge** | 29.00 | 6.76 | 137.1 | 3.72 |
| **Transfer to Another Hospital** | 42.95 | 7.04 | 429.2 | 2.84 |

Table 3: Disposition / Outcome by Mean Age, Deprivation, ED LOS, and Triage Score

| **Deprivation Level** | **European (% total)** | **Maori (% total)** | **Mean ED LOS (minutes)** |
| --- | --- | --- | --- |
| **1** | 2080 (83.3%) | 227 (9.1%) | 275.2 |
| **2** | 2696 (84.9%) | 260 (8.2%) | 280.9 |
| **3** | 1963 (86.5%) | 237 (10.4%) | 298.2 |
| **4** | 5747 (81.1%) | 725 (10.2%) | 285.5 |
| **5** | 7092 (78.7%) | 1248 (13.8%) | 272.2 |
| **6** | 6699 (80.3%) | 1158 (13.9%) | 300.2 |
| **7** | 4065 (71.4%) | 1196 (21%) | 287.5 |
| **8** | 12 125 (75.4%) | 2844 (17.7%) | 319.7 |
| **9** | 13 787 (70.4%) | 3975 (20.3%) | 319.8 |
| **10** | 3949 (61%) | 2008 (31%) | 313.8 |

Table 4: Deprivation Level (least to greatest) by Ethnicity and ED LOS

| **Ethnicity** | **Number** | **Mean Age (CI 95%)** | **Mean Deprivation (CI 95%)** | **Mean ATS category (CI 95%)** | **Mean ED LOS (CI 95%)** |
| --- | --- | --- | --- | --- | --- |
| **Maori** | 13 939 | 29.89 (29.513-30.264) | 7.50 (7.464-7.537) | 3.364 (3.351-3.377) | 266.8 (262.9-270.7) |
| **European** | 60 601 | 46.85 (46.629-47.062) | 6.62 (6.599-6.639) | 3.327 (3.321-3.333) | 315.9 (313.6-318.1) |

Table 5: Demographics by ethnicity

European patients were more likely to identify a GP (7.9% of Europeans did not identify a GP, while 14.9% of Maori did not identify a GP).

Age appeared to be a confounding variable for ethnicity and length of stay, but further exploration of the data demonstrated that there were many confounding variables, and reliable conclusions could not be reached about drivers of differences with exploratory analysis of the raw data. Therefore, in-depth analysis was undertaken and a complex predictive model developed to determine the drivers of ED length of stay.
