# Supplementary material for: Emergency Department Length of Stay for Maori and European Patients in New Zealand
Source: West J Emerg Med. 2016 Jun 21;17(4):438–48. doi: 10.5811/westjem.2016.5.29957 (PMC4944800; doi:10.5811/westjem.2016.5.29957)
Supplement: Supplementary file 3 [file wjem-17-438-s003.docx]

Appendix 3

Initial Patient Presenting Complaint (as recorded by ED receptionist, triage nurse, or charge nurse), by Ethnicity

|  |  | | | |  |  |
| --- | --- | --- | --- | --- | --- | --- |
| Presenting  Complaint | European | Maori | Pacific Is. | Asian | Other | Not Stated |
|  |  |  |  |  |  |  |
| other | 23676 | 5550 | 1017 | 1010 | 235 | 235 |
| Abdominal_pain | 4394 | 910 | 143 | 240 | 51 | 36 |
| Atrial fibrillation | 194 | 13 | 1 | 2 | 0 | 0 |
| Asthma | 509 | 292 | 56 | 23 | 4 | 1 |
| Back_pain | 812 | 140 | 27 | 22 | 12 | 5 |
| Cellulitis | 308 | 50 | 11 | 6 | 1 | 0 |
| Chest_infection | 253 | 72 | 7 | 10 | 1 | 0 |
| Chest_pain | 4088 | 620 | 110 | 121 | 34 | 41 |
| Collapse | 1326 | 174 | 35 | 45 | 6 | 12 |
| Confusion | 176 | 16 | 1 | 1 | 0 | 0 |
| Cough | 211 | 103 | 27 | 19 | 4 | 1 |
| CVA (cerebrovascular accident) | 123 | 12 | 2 | 4 | 0 | 0 |
| D&V (diarrhoea & vomiting) | 115 | 28 | 4 | 6 | 1 | 1 |
| Decreased_Mobility | 127 | 9 | 1 | 2 | 0 | 1 |
| Dehydration | 91 | 13 | 3 | 6 | 0 | 2 |
| Diahorrea | 167 | 24 | 7 | 9 | 1 | 2 |
| Epistaxis | 215 | 48 | 10 | 8 | 4 | 1 |
| ETOH (alcohol) | 216 | 53 | 8 | 6 | 0 | 1 |
| Facial_injuries | 229 | 57 | 6 | 7 | 4 | 3 |
| Fall | 763 | 61 | 10 | 10 | 3 | 5 |
| Fever | 530 | 237 | 42 | 80 | 18 | 5 |
| For_crisis | 224 | 87 | 2 | 2 | 0 | 2 |
| Foreign_body | 625 | 86 | 13 | 23 | 4 | 11 |
| Fracture | 101 | 25 | 1 | 3 | 1 | 0 |
| Haematuria | 160 | 15 | 2 | 3 | 0 | 1 |
| Head_injury | 789 | 230 | 39 | 25 | 5 | 11 |
| Headache | 721 | 169 | 38 | 41 | 8 | 5 |
| Hyperglycaemia | 76 | 17 | 6 | 1 | 0 | 0 |
| Hypertension | 117 | 13 | 2 | 7 | 0 | 1 |
| Hypoglycaemia | 72 | 9 | 6 | 3 | 2 | 0 |
| Infection | 460 | 145 | 29 | 12 | 2 | 5 |
| Injured | 3729 | 880 | 158 | 116 | 32 | 24 |
| MBA (motorbike accident) | 142 | 27 | 0 | 3 | 0 | 1 |
| MVA (motor vehicle accident) | 528 | 127 | 18 | 43 | 6 | 4 |
| Neck_pain | 137 | 24 | 6 | 6 | 1 | 0 |
| Overdose | 694 | 137 | 12 | 10 | 1 | 3 |
| Pain | 793 | 170 | 42 | 38 | 10 | 5 |
| Palpitations | 404 | 39 | 2 | 15 | 3 | 7 |
| Personal | 76 | 27 | 2 | 3 | 2 | 0 |
| Pneumonia | 96 | 22 | 8 | 1 | 0 | 0 |
| Post_op | 554 | 79 | 12 | 12 | 8 | 3 |
| PR (rectal)_bleed | 302 | 35 | 6 | 10 | 4 | 5 |
| PV (vaginal)_bleed | 358 | 119 | 21 | 35 | 4 | 1 |
| Query_CVA (cerebrovascular accident) | 190 | 13 | 5 | 0 | 0 | 0 |
| Query_pneumonia | 74 | 26 | 4 | 4 | 0 | 0 |
| Query_TIA (transient ischaemic attack) | 186 | 18 | 1 | 4 | 1 | 2 |
| Query_UTI (urinary tract infection) | 126 | 18 | 5 | 4 | 3 | 1 |
| Rash | 295 | 142 | 32 | 33 | 8 | 9 |
| Return | 360 | 83 | 10 | 24 | 7 | 3 |
| Revisit | 783 | 179 | 33 | 29 | 9 | 6 |
| Seizure | 506 | 232 | 10 | 8 | 1 | 2 |
| SOB (shortness of breath) | 3197 | 922 | 168 | 99 | 30 | 13 |
| Sore_throat | 135 | 61 | 23 | 23 | 1 | 2 |
| SVT (supraventricular tachycardia) | 82 | 49 | 0 | 3 | 0 | 1 |
| Tachycardia | 78 | 11 | 1 | 1 | 0 | 1 |
| TIA (transient ischaemic attack) | 92 | 9 | 1 | 0 | 1 | 0 |
| Toothache | 222 | 96 | 13 | 11 | 3 | 2 |
| Unwell | 3644 | 944 | 189 | 206 | 59 | 8 |
| Urinary_retention | 207 | 9 | 2 | 2 | 1 | 3 |
| UT (urinary tract infection) | 160 | 25 | 7 | 6 | 1 | 2 |
| Vertigo | 89 | 6 | 4 | 3 | 1 | 0 |
| Vomiting | 494 | 132 | 30 | 56 | 19 | 4 |
